# Humeral head osteonecrosis in an adolescent amateur swimming athlete: a case report

**DOI:** 10.1186/1758-2555-4-39

**Published:** 2012-10-18

**Authors:** Jianlin Zuo, Hirotaka Sano, Nobuyuki Yamamoto, Yoshimasa Sakoma, Nobuhisa Shinozaki, Yoshiaki Itoigawa, Rei Omi, Eiji Itoi

**Affiliations:** 1 Department of Orthopaedic Surgery, Tohoku University School of Medicine, 1-1 Seiryo-machi Aoba-ku, Sendai, 980-8574, Japan; 2 Department of Orthopaedic Surgery, China-Japan Union Hospital of Jilin University, 126 Xiantai Street, Changchun, 130033, China

**Keywords:** Osteonecrosis, Shoulder, Adolescent, Pathogenesis, MRI

## Abstract

Osteonecrosis of the humeral head in an adolescent without clear pathogenesis has not been reported in the literature. In this case report, we present such a case of humeral head osteonecrosis in a 15-year-old adolescent. The lesion was located at the subchondral area of the medial part of the humeral head with characteristic appearances on MRI. The shoulder was immobilized in a sling until the pain disappeared, and the patient was told to refrain any kind of sport activities. Bone remodeling was noted five months after the first visit, and it took 2 years for the lesion to be totally healed.

## Background

Osteonecrosis of the humeral head has been well documented in adults. Steroid induced and sickle-cell disease related humeral head osteonecrosis also have been reported in children and adolescents. David et al [1] reported a group of 138 patients of sickle-cell disease related humeral head osteonecrosis. In his group, the youngest age was 5 years. In 2004, Pateder et al [2] reported a case of humeral head osteonecrosis in a thirteen-year-old adolescent occurring after shoulder stabilization for the treatment of recurrent shoulder dislocation. Because the operation was done in an open manner by the deltopectoral approach, the authors suspected the possible damage to the anterolateral ascending branch of the anterior circumflex humeral artery being responsible for the osteonecrosis of humeral head. Wang et al [3] reported a case of revascularization of the humeral head after osteonecrosis secondary to a fracture dislocation in a ten-year-old boy. To our knowledge, osteonecrosis of the humeral head in an adolescent without clear pathogenesis has not been reported in the literature. In this case report, we present such a case of humeral head osteonecrosis in a 15-year-old adolescent. The lesion totally healed 2 years after the occurrence without any surgical intervention.

## Case presentation

A 15-year-old male adolescent, an amateur swimming athlete, visited our clinic. His chief complaint was pain in the right shoulder when swimming. He first felt pain in his right shoulder 5 years before, but the pain disappeared just after a one-day rest, so he did not see a doctor. Four years after the incidence, the pain recurred during swimming. It was still mild but occasional from then on. Several days prior to his first visit to our clinic, the pain suddenly got worse and he consulted an orthopaedic doctor, who took x-rays and MRI of his right shoulder and then referred the patient to our clinic.

His pain was located at the lateral portion of the right shoulder. The intensity of pain assessed by visual analog scale was 00/100 at rest, 25/100 during motion, and 00/100 at night. There was no muscle atrophy and no positive impingement sign. There was a slightly limited range of motion of the right shoulder: flexion, 150 degrees; abduction, 120 degrees; external rotation, 75 degrees; and internal rotation, T4.

**Figure 1:**
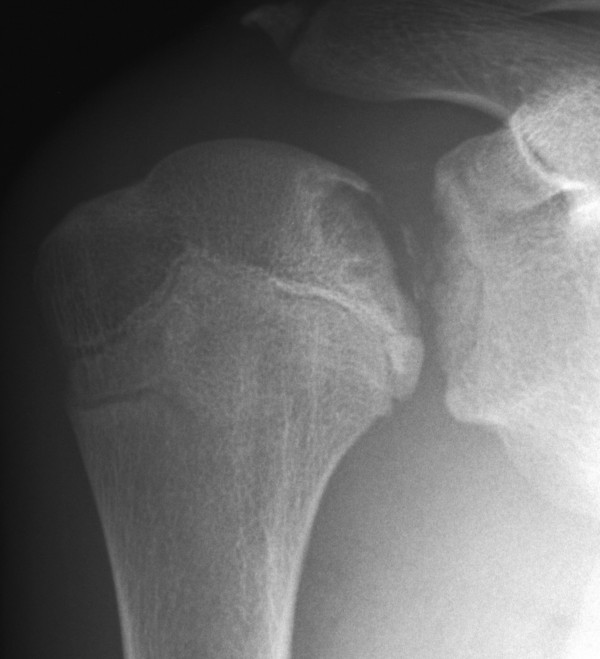
An anteroposterior plain radiograph (2007-5-25) shows the lesion became larger and a sclerotic band appeared around the lesion.

**Figure 2:**
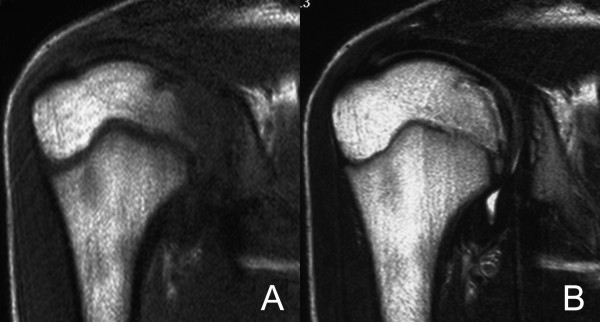
A coronal oblique T1-weighted magnetic resonance image of the shoulder (2007-5-25) (spin-echo sequence with a repetition time of 626 msec and an echo time of 13 msec) shows a focal low signal area in the medial part of humeral head (A), and a coronal oblique T2-weighted magnetic resonance image of the shoulder (2007-5-25) (spin-echo sequence with a repetition time of 3775 msec and an echo time of 90 msec) shows an irregular low signal band on the corresponding part of humeral head (B).

**Figure 3:**
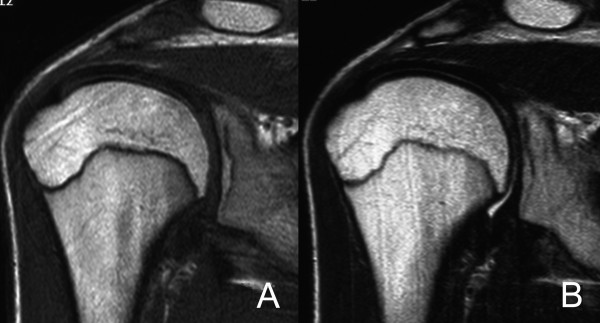
A coronal oblique T1-weighted magnetic resonance image of the shoulder (2008-6-30) (spin-echo sequence with a repetition time of 500 msec and an echo time of 13 msec) shows normalization of the epiphyseal marrow signal (A), and a coronal oblique T2-weighted magnetic resonance image of the shoulder (2008-6-30) (spin-echo sequence with a repetition time of 3762 msec and an echo time of 90 msec) shows normalization of the epiphyseal marrow signal (B).

**Figure 4:**
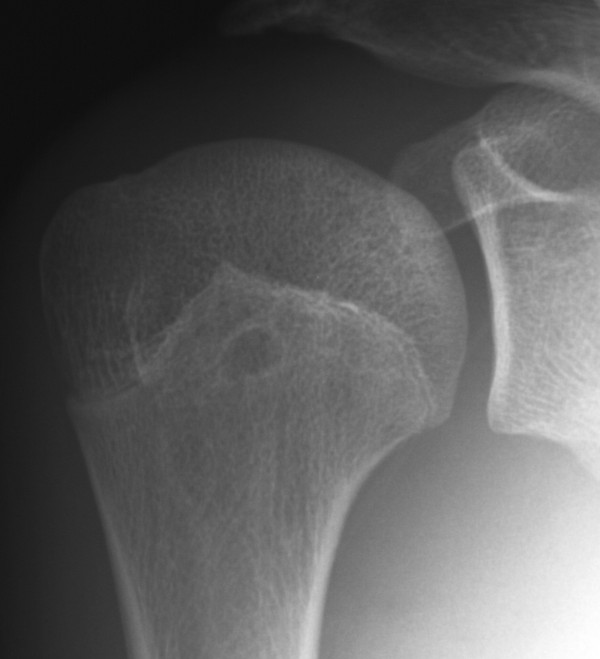
An anteroposterior plain radiograph (2009-3-31) shows the lesion had been totally healed.

An anteroposterior radiograph showed osteolysis of the subchondral area of the medial part of the humeral head. MRI (T1- and T2-weighted images) revealed an inhomogeneous low signal area in the medial part of the humeral head with high signal in the joint space on T2-weighted image. Although administration of any kind of steroid, history of sickle-cell disease and other risk factors for osteonecrosis were denied, based on the clinical findings, we primarily established a diagnosis of osteonecrosis of the right humeral head. According to the classification system of Cruess [4], the lesion was classified as stage 3, which was characterized by the crescent sign (subchondral fracture). We talked to the patient to stop swimming or any kind of sport activities. The shoulder was immobilized in a sling until the pain disappeared. We decided to observe the patient, expecting the lesion would be remodeled, but also with an option of core decompression in case it would not be remodeled. However, on May 28, 2007, when the patient revisited us, the plain x-rays and MRI both showed that the lesion became clearer in shape and a little larger in size. A sclerotic band appeared surrounding the lesion on the x-ray (Figure 1). The low signal area on MRI scan became homogeneous and the margin became clear (Figure 2). The patient complained only of slight motion pain with the range of motion well preserved. Due to a rapid aggravation of the lesion, a malignant disease was also suspected and bone scintigraphy was performed, but no abnormal findings were observed. We talked to the patient that a biopsy might be necessary if the lesion would still expand in 2 months. On the following visit, the pain subsided completely with a full range of motion of the right shoulder. Both plain x-ray and MRI showed that bone remodeling had started and the lesion had become smaller. These findings confirmed the diagnosis of osteonecrosis. Still the patient was not permitted to perform any sport activities. On December 25, 2007, new MRI scans revealed that the lesion had become even smaller and there was only a low signal band left on several slices in the coronal oblique view. On June 30, 2008, the healing progressed even further, and abnormal signal had disappeared on all but 2 slices, on which only a thin and vague low signal line could be observed on the posteromedial subchondral area (Figure 3). Because the healing process was almost completed, the patient was permitted to start swimming in a gradual manner. On the latest visit of March 31, 2009, the lesion was totally healed with normal appearance on plain radiograph (Figure 4). There was no pain and the range of motion was full.

## Discussion

Although some theories have been proposed to interpret the pathogenesis of osteonecrosis, the theory of blood supply interruption by various initiative conditions is now the most commonly accepted one. In traumatic cases, the blood vessel that supplies certain part of the humeral head may be destroyed directly, whereas in non-traumatic cases, the conditions such as corticosteroid use, sickle-cell disease, alcoholism, dysbarism, Gaucher’s disease, and other systemic conditions can lead to a hypercoagulable state and then in turn to thrombosis or emboli to block the vessel and at last lead to the osteonecrosis. Gerber et al [5] studied the intraosseous distributions of arteries in twenty-nine shoulder specimens from fresh cadavers. He found that the anterolateral ascending branch of the anterior circumflex artery contributed most part (anterior and superior) of the blood supply of the humeral head. The posterior circumflex artery, on the other hand, vascularized only the posterior portion of the greater tuberosity and a small posteroinferior part of the head. Although anastomoses between the different arteries were abundant, vascularization of all the humeral head was possible only through the anterolateral branch of the anterior circumflex artery. The arcuate artery, which is the intraosseous part of the anterolateral branch of the anterior circumflex artery, distributed along the metaphyseal side of the epiphyseal plate. Small branches crossed the plate and were given off to the epiphysis and cartilage at right angles to the arcuate vessel. The subchondral bone of the humeral head is especially vulnerable to thrombotic and embolic phenomenon, because the arterioles in this area become sinusoids that turn 180° to return to the intraosseous circulation [5,6]. The failure of the small branches that vascularize the inferior part of the epiphysis may be blamed to the necrosis in this case and the reconstruction of these branches should contribute to the revive of the necrotic part of the epiphysis. External revascularization may develop via periosteal reaction at the inferior joint capsule, which may also contribute to the remolding of the humeral head. Unlike the femoral head, there have been no studies on the blood supply specificities of the humeral head on skeletally-immature subjects. Future studies focusing on this issue need to be undertaken. As previously noted by Cruess [7] and Neer [8], the osteonecrotic lesion is typically located at the site of glenohumeral contact in approximately 90 degrees of shoulder abduction. This has been shown to be the position of maximum force transmission across the glenohumeral joint [9]. The lesion in this case was observed exactly at this location. Furthermore, Bergmann et al [10] measured in vivo glenohumeral contact forces using an instrumented shoulder implant with telemetric data transmission. He found that the contact force remained below 100% BW (percent body weight) for most activities of daily living. It ranged up to 130% BW in arm positions close to the limits of motion or when acting against external resistance. Because the latter situation is common during swimming, we speculate that the high contact force might play a role in causing the lesion.

As osteonecrosis of the femoral head, the lesion in the humeral head also have no trend toward natural healing in adults. Hattrup et al [11] reported a group of 151 patients with 200 shoulders affected by humeral head osteonecrosis. Of the 200 shoulders, subsequent surgery was required in 102. Of the 60 shoulders that had been treated conservatively and followed up successfully for an average of 8.6 years, there was moderate to severe pain in 31 shoulders. However, the natural history of osteonecrosis of the humeral head in this adolescent case was quiet different. During the 2-year period, MRI examination was repeated 6 times. They revealed that the healing process (bone formation) was first initiated on the distal side and then progressed proximally. Meanwhile, the bone remodeling accomplished earlier on the anterior side than on the posterior side. According to the vascularization area of the ascending branch of the anterior circumflex artery, this vessel is likely to contribute to the most part of the reviving procedure. The natural healing in this case took 2 years to be completed, which was consistent with those reported by other authors [2,3].

## Conclusions

An adolescent with humeral head osteonecrosis has a good chance of natural recovery with proper protection from vigorous activities.

## Consent

Written informed consent was obtained from the patient for publication of this Case report and any accompanying images. A copy of the written consent is available for review by the Editor-in-Chief of this journal.

## Competing interests

The authors declare that they have no competing interests.

## Authors' contributions

All authors co-wrote the paper and discussed the results for the manuscript preparation. All authors have read and approved the final manuscript.
